# Draft Genome Sequences of 12 Clostridium tyrobutyricum Strains Isolated from Raw Milk and Cheese

**DOI:** 10.1128/MRA.00735-21

**Published:** 2021-09-30

**Authors:** L. Podrzaj, J. Burtscher, K. J. Domig

**Affiliations:** a BOKU—University of Natural Resources and Life Sciences Vienna, Vienna, Austria; University of Rochester School of Medicine and Dentistry

## Abstract

Clostridium tyrobutyricum is recognized as the main causative agent of late blowing defect—severe spoilage of hard and semihard cheeses. In this work, we present the draft genome sequences of 12 *C. tyrobutyricum* strains isolated from raw milk and cheese.

## ANNOUNCEMENT

Clostridium tyrobutyricum is an endospore-forming anaerobic bacterium recognized as the main causative agent of late blowing defect (LBD)—severe spoilage of hard and semihard cheeses, leading to great financial losses for the dairy industry (1). Clostridial endospores, which contaminate raw milk, can germinate and outgrow to vegetative cells during cheese ripening, leading to butyric fermentation and consequently causing deformation of cheese loafs, as well as sensory changes. However, not all *C. tyrobutyricum* strains seem to be equivalent in their spoilage potential (2–7). Here, we report the draft genome sequences of 12 *C. tyrobutyricum* strains, including 5 strains (Cl_64, Cl_80, Cl_82, Cl_84, and Cl_117) isolated from raw milk samples (8); 4 strains (Cl_171, Cl_188, Cl_238, and Cl_239) isolated from spoiled cheese samples (9); 1 strain (Cl_14; synonym DSM 663) obtained from the German Collection of Microorganisms and Cell Cultures (DSMZ, Braunschweig, Germany), originally isolated from Emmental cheese; 1 strain (Cl_29) isolated from strain NIZO BZ15, which was isolated from spoiled cheese and was obtained from the culture collection of NIZO food research (Ede, The Netherlands); and 1 strain (Cl_52) isolated from strain FAM25158 from the Agroscope Culture Collection (Liebefeld, Switzerland), originally isolated from Emmental cheese. Species assignment of the strains was conducted based on 16S rRNA gene sequencing, following a protocol detailed previously (6, 9), and confirmed by matrix-assisted laser desorption ionization–time of flight mass spectrometry (MALDI-TOF MS) using a Microflex LT instrument and MALDI Biotyper software (Bruker Daltonik, Bremen, Germany) according to the manufacturer’s instructions.

Frozen stocks (−80°C in 20% glycerol) of *C. tyrobutyricum* strains were streaked onto reinforced clostridial agar (RCA) (Merck, Darmstadt, Germany), and single colonies were grown anaerobically at 37°C overnight in reinforced clostridial medium (RCM) (4). Genomic DNA was obtained from bacterial pellets using the peqGOLD bacterial DNA kit (Peqlab, Erlangen, Germany) according to the manufacturer’s instructions, except that the initial lysis step using lysozyme was performed at 37°C for 30 min, and the DNA was eluted with 30 μl elution buffer and incubated for 5 min at 70°C without agitation in an Eppendorf thermomixer, followed by an additional elution step. Library preparation and sequencing were performed by Novogene (Cambridge, UK). Genomic libraries were prepared using a NEBNext DNA library prep kit (New England BioLabs, USA), with sequencing on the Illumina NovaSeq 6000 platform (2×150-bp reads). FastQC v0.11.9 (10) was used to control the raw read quality, Trimmomatic v0.3 (11) was used to remove adapter sequences and sequences with quality scores of <25, and SPAdes v3.15.2 (12) was used for read assembly, employing k-mers ranging from 23 to 123. The assembly metrics were calculated using QUAST v5.0.2 (13), discarding contigs less than 200 bp using PRINSEQ (14). All reads were mapped back to the assembly using BWA-MEM v0.7.17 (15) and were sorted using SAMtools v1.9 (16) to determine the average genome coverage values. The draft genome sequences were annotated using the NCBI Prokaryotic Genome Annotation Pipeline (PGAP) v5.2 (17). Unless specified otherwise, default parameters were used for all software. The raw read statistics, genome assembly metrics, and accession numbers are shown in Table 1.

**TABLE 1:** Genome features and metadata of twelve Clostridium tyrobutyricum strains

Strain ID	Isolate source	Isolation country	Yr of isolation	Genome size (bp)	No. of raw reads	No. of contigs	G+C content (%)	*N*_50_ (bp)	Coverage (×)	Total no. of genes	No. of CDSs^*a*^	No. of tRNAs	No. of rRNAs	GenBank accession no.	SRA accession no.^*b*^
Cl_14 (DSM 663)	Emmental cheese	Unknown	Before 1964	3,144,217	2,909,087	106	30.72	98,614	80	3,223	3,114	35	3	JAHHVA000000000	SRR12960712
Cl_29	Strain NIZO BZ15 (originally isolated from spoiled cheese)	Unknown	1970	3,080,479	2,746,728	76	30.83	91,495	75	3,099	3,006	37	4	JAHHUZ000000000	SRR12960711
Cl_52	Strain FAM25158 (originally isolated from Emmental cheese)	Unknown	1966	3,076,120	3,976,862	152	30.49	71,003	116	3,080	2,966	42	2	JAHHUY000000000	SRR12960708
Cl_64	Raw milk	Austria	2016	3,166,217	3,244,271	110	31.03	76,461	88	3,220	3,096	46	2	JAHHUX000000000	SRR12960707
Cl_80	Raw milk	Austria	2016	3,079,608	5,686,507	118	30.91	78,420	80	3,155	3,065	34	3	JAHHUW000000000	SRR12960706
Cl_82	Raw milk	Austria	2016	2,998,277	3,352,985	145	30.81	52,932	96	3,048	2,941	45	4	JAHHUV000000000	SRR12960705
Cl_84	Raw milk	Austria	2016	3,043,301	3,168,618	115	30.87	81,581	89	3,083	2,964	52	3	JAHHUU000000000	SRR12960704
Cl_117	Raw milk	Austria	2016	3,094,383	3,494,568	117	31.04	74,689	90	3,167	3,044	46	4	JAHHUT000000000	SRR12960703
Cl_171	Semihard cheese	Austria	2015	3,037,827	2,616,749	58	30.88	140,508	73	3,058	2,953	36	3	JAHHUS000000000	SRR12960702
Cl_188	Cheese-sausage mixture	Austria	2015	3,005,637	3,519,200	56	30.74	164,768	113	2,990	2,883	40	3	JAHHUR000000000	SRR12960701
Cl_238	Hard cheese	Austria	2015	3,225,706	4,304,866	101	30.79	89,274	89	3,238	3,127	30	4	JAHHUQ000000000	SRR12960710
Cl_239	Hard cheese	Austria	2015	3,319,585	3,398,168	107	30.83	84,140	94	3,361	3,228	48	4	JAHHUP000000000	SRR12960709

a CDSs, coding sequences with proteins.

b SRA, Sequence Read Archive.

### Data availability.

The draft genome sequences and raw reads are available in GenBank under the BioProject accession number PRJNA673079.
